# Bio-Inspired Molecularly Imprinted Polymer Electrochemical Sensor for Cortisol Detection Based on O-Phenylenediamine Optimization

**DOI:** 10.3390/biomimetics8030282

**Published:** 2023-07-01

**Authors:** Minwoo Kim, Daeil Park, Joohyung Park, Jinsung Park

**Affiliations:** Department of Biomechatronic Engineering, College of Biotechnology and Bioengineering, Sungkyunkwan University, Suwon 16419, Republic of Korea; yegrina93@gmail.com (M.K.); pdi9907@gmail.com (D.P.)

**Keywords:** biomimic, cortisol, molecularly imprinted polymer, O-PD optimization, electrochemistry

## Abstract

This paper presents a comprehensive investigation of the various parameters involved in the fabrication of a molecularly imprinted polymer (MIP) sensor for the detection of cortisol. Parameters such as monomer concentration, electropolymerization cycles, pH, monomer–template ratio, template removal technique, and rebinding time were optimized to establish a more consistent and effective method for the fabrication of MIP sensors. Under the optimized conditions, the MIP sensor demonstrated a proportional decrease in differential pulse voltammetry peak currents with increasing cortisol concentration in the range of 0.1 to 100 nM. The sensor exhibited excellent sensitivity, with a limit of detection of 0.036 nM. Selectivity experiments using a non-imprinted polymer sensor confirmed the specific binding affinity of the MIP sensor for cortisol, distinguishing it from other steroid hormones. This study provides crucial insights into the development of a reliable and sensitive strategy for cortisol detection using O-PD-based MIPs. These findings laid the foundation for further advancements in MIP research.

## 1. Introduction

The rapid growth of various industries has significantly contributed to high stress levels [1]. Additionally, amidst the ongoing COVID-19 pandemic, stress has become even more pervasive, making effective stress management a critical concern for individuals worldwide. Cortisol, a representative stress hormone, is crucial for regulating various bodily functions, including metabolism, immune responses, and stress management [2,3,4,5]. The inadequate management of cortisol levels can lead to several adverse symptoms such as fatigue, mood swings, and a weakened immune system [6]. Therefore, quantifying cortisol levels in a simple and effective manner can help individuals maintain their physical and mental well-being.

Accordingly, several methods have been developed for cortisol detection; however, their accuracy and ease of use have some limitations. Traditional methods such as radioimmunoassay [7], enzyme-linked immunosorbent assay [8,9], and chromatographic techniques [10,11] are expensive, time consuming, and require specialized equipment and personnel. Moreover, state-of-the-art measurement methods, such as Raman spectroscopy [12,13], localized surface plasmon resonance [14,15], and quartz crystal microbalance [16], have been introduced to determine cortisol levels. Although these analytical methods exhibit excellent sensitivity, they are expensive and require trained personnel. Researchers have explored the use of molecularly imprinted polymers (MIPs) to overcome these challenges in detecting cortisol levels [17]. MIPs are a bio-inspired technique that mimics the morphological binding observed in antigen–antibody responses [18]. It can be designed to selectively bind to cortisol, enabling a more specific and efficient approach for cost-effective cortisol detection [19,20,21]. Although MIP technology shows promise for cortisol detection, much research remains to be conducted. One challenge is that the methods of using MIPs to detect cortisol vary widely among studies. This is because the conditions and optimizations used to produce MIPs can differ, leading to variations in their selectivity and sensitivity for cortisol detection [20,22,23,24]. By establishing more consistent methods, MIPs can become valuable for the accurate and efficient monitoring of cortisol levels, improving stress management, and promoting better overall health.

In this study, cortisol was effectively detected using a bio-inspired MIP optimized with the commonly used monomer O-Phenylenediamine (O-PD). The optimization parameters and values were compared with those obtained in previous studies. Different O-PD concentrations, electropolymerization cycles, pH values, and monomer–template ratios were tested to optimize the cortisol MIP sensor. Furthermore, various template-removal methods and rebinding times were tested. This comprehensive approach allowed us to carefully consider and optimize the response according to the changes in these parameters, which have not been thoroughly explored in existing O-PD-based MIP studies. The optimized MIP sensor was compared with non-imprinted polymer (NIP) to confirm its sensitivity, and similar biomolecules (cortisone, corticosterone, progesterone, and triamcinolone) were tested using the optimized MIP to ensure selectivity [14]. This research will make significant contributions to the fundamental study of cortisol detection.

## 2. Materials and Methods

### 2.1. Chemicals and Materials

Chemicals, including sodium phosphate dibasic, sodium phosphate monobasic monohydrate, sodium acetate, hydrocortisone, o-phenylenediamine, phosphate-buffered saline (PBS, 10X, pH 7.4), acetic acid (99.7%), sodium hydroxide solution (50%), cortisone, corticosterone, progesterone, triamcinolone acetonide, potassium hexacyanoferrate (II) and (III), and potassium chloride were purchased from Sigma-Aldrich (Darmstadt, Germany). Ethyl alcohol (99.9%), methyl alcohol (99.8%), and sulfuric acid (98%) were purchased from Daejung Chemicals (Siheung-si, Republic of Korea). In the present study, we used ultrapure distilled water with a resistivity of 18.2 MΩ·cm. The screen-printed carbon electrodes (SPCEs) were obtained from DropSens (DRP-C110, DropSens, Oviedo, Spain).

### 2.2. Fabrication of the Molecularly Imprinted Polymer(MIP) Cortisol Sensors

First, SPCEs were preoxidized using 0.1 M phosphate buffer solution (PB, pH 7.0) from 0 to 1.3 V at a scanning rate of 0.1 V/s for 20 cycles to electrochemically clean the electrode surface. Subsequently, the cleaned SPCEs were oxidized at 2.0 V for 30 s in 0.1 M PBS and immediately rinsed using distilled water.

The MIP films were electropolymerized onto the functionalized SPCE in 0.01 M PBS solution (pH 7.4) or acetate buffer (pH 5.2) containing a mixture of 3.5 mM o-PD, 0.5 mM cortisol templates, and 0.1 M KCl. Electropolymerization was performed under the following conditions: a scan rate of 0–1 V, E-step of 4 mV, scan of 30 cycles, and scan rate of 50 mV/s. The fabricated MIP films were washed immediately with distilled water.

After the electropolymerization process, cortisol templates were removed from polymer matrix via overoxidation with 0.01 M PBS under the following conditions: a scan range of −0.2–0.8 V, an E-step of 5 mV, a N scan of 25 cycles; and a scan rate of 50 mV/s. After overoxidation, all electrodes were immediately rinsed with 10 mM PBS and left to dry in a vacuum chamber for 1 h. Non-imprinted polymers (NIPs) were prepared using the same method but without cortisol in the polymerization solution.

### 2.3. Electrochemical Apparatus and Measurements

Cyclic voltammetry (CV) and differential pulse voltammetry (DPV) were performed using an IviumStat potentiostat and analyzed using IviumStat software (Ivium Technologies, Eindhoven, Netherlands).

To characterize sensor performance, the MIP sensors were first incubated with various concentrations of cortisol in 1 × PBS for 10 min. After incubation, all the electrodes were washed immediately with 10 mM PBS and left to dry overnight in a vacuum chamber. Thereafter, the CV and DPV measurements were performed in 80 μL containing 10 mM PBS and 5 mM [Fe(CN6)]^3−/4−^ as the redox probe at room temperature. The following CV parameters were used: a scan range of −0.35–0.8 V for measuring the redox reaction on the electrode; an E-step of 5 mV; and a scan rate of 50 mV/s. The following DPV parameters were used: a potential range of 0–0.8 V, a pulse amplitude of 50 mV/s, a pulse time of 10 ms, an E-step of 4 mV, and a scan rate of 50 mV/s.

## 3. Results and Discussion

### 3.1. Cortisol Detection Strategy

**Scheme 1:**
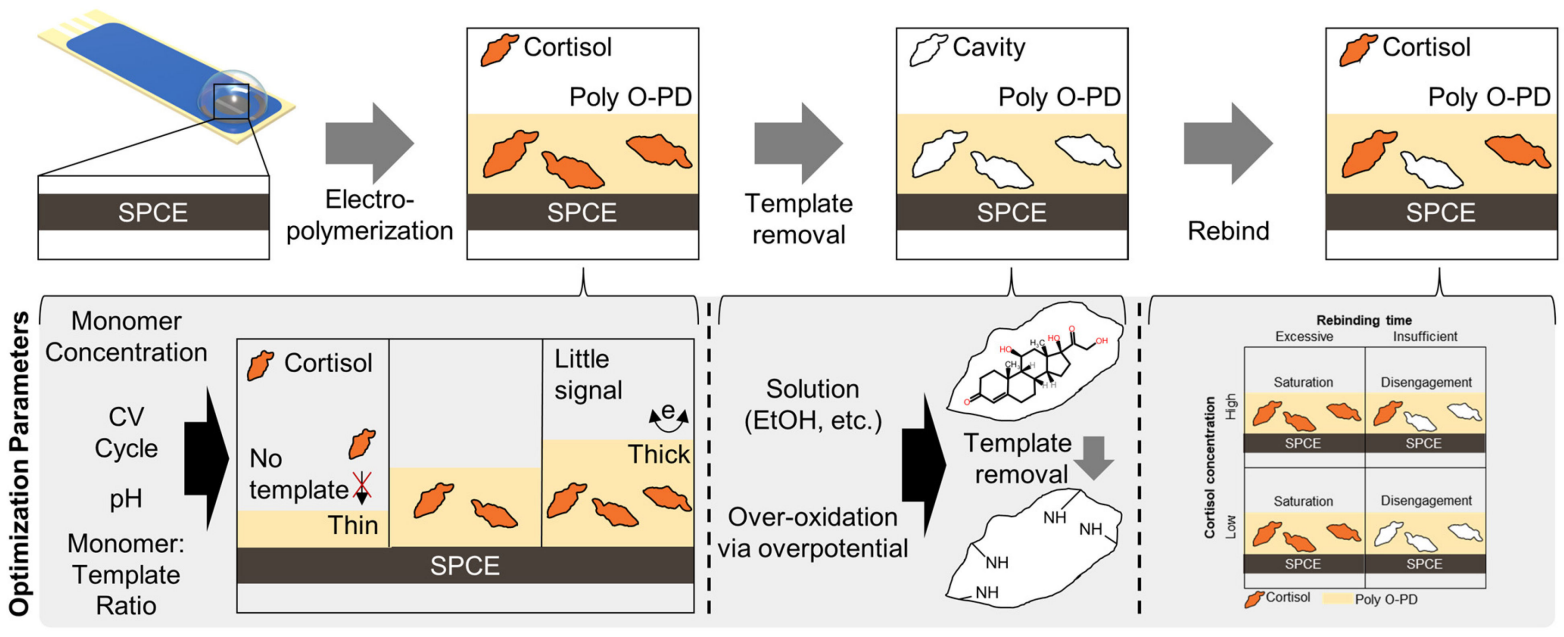
Schematic illustration of MIP electrode fabrication and optimization parameters.

To effectively detect cortisol, we utilized a bio-inspired MIP system that mimics antigen–antibody binding. MIP electrode fabrication typically involves three stages: electropolymerization, template removal, and rebinding (Scheme 1). During electropolymerization, a thin layer of MIP is deposited on the surface of the electrode by applying an electric potential. The target molecule was added to the solution containing the monomer during electropolymerization to create specific binding sites within the MIP for cortisol. Optimizing parameters such as monomer concentration, CV cycles, pH, and monomer–template ratio is crucial for achieving an appropriate thickness of the MIP layer, which is essential for creating effective binding sites for cortisol [19]. After electropolymerization, the template molecule, cortisol, was removed, leaving behind specific binding sites for cortisol. Template removal can be achieved using various methods, including solvent extraction and overoxidation. In the rebinding stage, the MIP electrode was exposed to a solution containing cortisol, and the binding between cortisol and MIP was measured using CV and differential pulse voltammetry (DPV) [24]. These three stages were optimized to tailor the MIP electrode to a specific target molecule, cortisol, with high selectivity and sensitivity.

Previous MIP sensors fabricated using O-PDs were comprehensively investigated, and the optimization parameters are listed in Table 1 to facilitate the selection of appropriate parameters. Template and monomer concentrations are critical factors for the fabrication of MIPs. The monomer concentration influences the thickness and morphology of the MIP layer as well as the number of binding sites created for the target molecule. Similarly, the template concentration affects the formation of the template–monomer complex and the imprinting effect. The optimal template and monomer concentrations are crucial for achieving the best imprinting effect and selectivity. In previous studies, the range of template concentration was 0.1 mM to 120 mM; however, numerous studies reported the range as 1–20 mM [25,26,27,28,29,30]. The monomer concentration range was smaller than that of the template, and most studies were optimized for 5 mM O-PD. The number of electropolymerization CV cycles affected the thickness of the MIP film, which is another crucial factor for the MIP sensor. Studies range between 6 and 30 cycles to form an MIP film of adequate thickness. The pH of the polymerization solution affects the ionization state of functional monomers, which can influence the formation of binding sites in MIP. Therefore, careful selection of pH is necessary to ensure optimal MIP sensor performance. Through this review, parameters such as template concentration, monomer concentration, number of CV cycles, and solution pH were selected for optimization in the electropolymerization stage.

Other factors in the electropolymerization stage of the MIP sensor fabrication process, such as the scan rate and potential range of CV, have been reported to be consistent across different experiments [31]. The scan rate, which is the speed at which the potential of the working electrode is swept from the initial to final potential per unit time, was set to 50 mV/s in most studies. A higher scan rate could lead to a higher rate of electrodeposition but might also result in less stable and less uniform polymer films. On the other hand, a lower scan rate could result in a more uniform film but might take a longer time to deposit the polymer layer. Therefore, 50 mV/s is commonly used as a compromise between the deposition rate and quality of the resulting polymer films. The potential range of the CV is dependent on the redox potential of the analyte, electrode material, and supporting electrolyte. The reduction and oxidation potential of O-PD is about −0.4–−0.5 V and 0.8–0.9 V (vs. Ag/AgCl), respectively. As result, most studies use a range within 0~0.8 V to form O-PD film via oxidation. Similar to other studies, the scan rate and potential range were maintained at 50 mV/s and 0~0.8 V, respectively.

In the template removal stage, the extraction solution should efficiently dissolve or extract template molecules from the polymer matrix without damaging the structure of the imprinted cavities [32]. In addition, the extraction method should be sufficiently gentle to prevent the formation of cracks or defects in the MIP film. The extraction solution and method differed from those in previous studies; therefore, optimization of the extraction solution and method was conducted. Finally, in the rebinding stage, the rebinding time is the time required for the target molecule to bind to the MIP [33]. The rebinding time can affect the sensitivity and selectivity of the MIP sensors. If the rebinding time is too short, the target molecule may not interact with MIP, leading to a weak signal. Conversely, if the rebinding time is too long, it may fully bind and reach an equilibrium, hindering the ability of the sensor to distinguish concentration differences. Therefore, rebinding time was selected as the optimization parameter in this study.

**Table 1:** Comparison of fabrication method of different electrochemical molecularly imprinted polymer (MIP) sensors.

Template	Monomer (mM)	pH	Scan Rate (mV/s)	# of Cycle	Voltage Range (V)	Extraction Solution	Extraction Time (min)	Rebind Time (min)	Reference
0.1 M Triclosan	6 (O-PD)	5.2	50	20	0–0.8	0.1 M NaOH	10	15	[34]
1 mM ATZ	5 (O-PD)	7.4	50	15	0–0.8	MeOH/Acetic acid (9:1 *v*/*v*)	N.A.	8	[30]
0.4 mM PMX	5 (O-PD)	5.2	50	20	−0.2–0.75	0.1 M NaOH	1	5	[35]
1 mM PFOs	10 (O-PD)	5.8	50	25	0–1	50% MeOH	20	26	[29]
20 mM Sorbitol	5 (O-PD)	5.2	50	30	0–0.8	DI	N.A.	10	[28]
0.4 mM 2,4-DCP	2 (O-PD)	5.2	50	10	0–1	EtOH	8	6	[36]
20 mM Glucose	5 (O-PD)	5.2	50	20	0–0.8	pH 5.2 Acet. Buffer /10 mM glucose	Short time	N.A.	[27]
0.12 M Aniline	3 (O-PD)	3	50	25	−0.1–1	DI/MeOH (6:4 *v*/*v*)	10	N.A.	[37]
10 mM GSH	5 (O-PD)	6.98	50	6	0–0.8	0.1 M NaOH	30	N.A.	[26]
0.5 mM Cortisol	5 (O-PD)	4	50	30	0–1	EtOH	40	N.A.	[38]
1–20 mM Cortisol	0.03–1 M (Pyrrole)	7.4	100	10	−0.2–0.9	PBS (overoxidation)	5–40 cycles	15	[25]
0.5 mM Cortisol	3.5 mM (O-PD)	5.2	50	30	0–1	PBS (overoxidation)	25 cycles	10	This work

ATZ = atrazine; PMX = pemetrexed; PFOs = perfluorooctanesulfonate; 2,4-DCP = 2,4-dichlorophenol; GSH = L-glutathione reduced; O-PD = O-phenylenediamine; NaOH = sodium hydroxide; MeOH = methyl alcohol; PBS = phosphate-buffered saline buffer; Acet. buffer = acetate buffer; DI = distilled water; N.A = not available.

### 3.2. Electropolymerization Parameters Optimization

**Figure 1:**
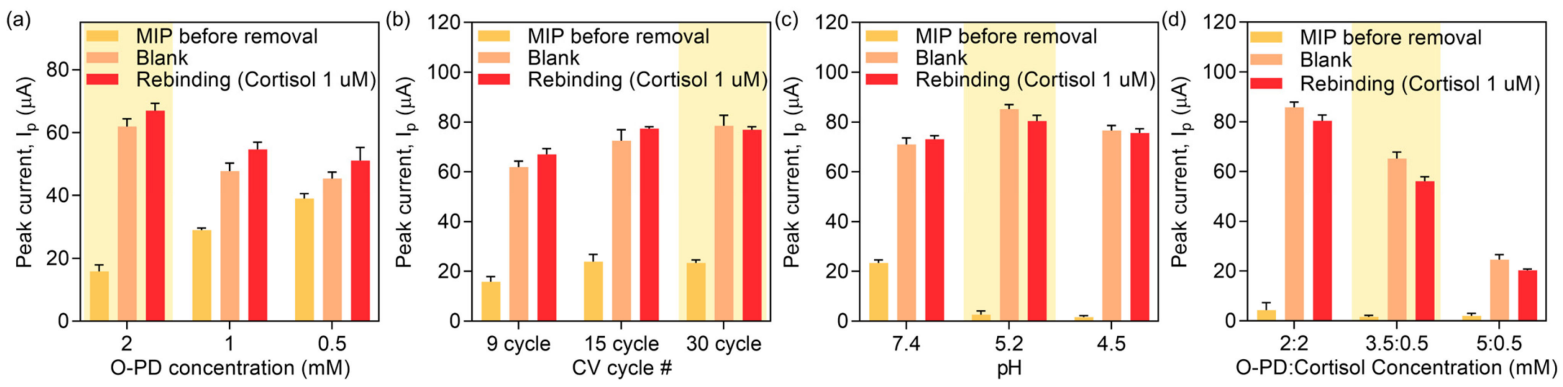
Optimization of electropolymerization parameters. (**a**) Influence of O-PD concentration on peak current of CV with a fixed cortisol concentration of 2 mM. (**b**) Peak current of CV at different number of electropolymerization cycles. (**c**) Peak current of CV at different pH values of electropolymerization solution. (**d**) Influence of O-PD and cortisol concentration on peak current of CV.

Electropolymerization parameters, such as monomer and template concentrations, pH, and number of cycles, were optimized to achieve the effective detection of cortisol (Figure 1). To optimize the monomer concentration, the template (cortisol) concentration was fixed at 2 mM and different concentrations of O-PD were used to fabricate the MIP sensor. The effect of O-PD concentration was evaluated by studying the CV response of the MIP electrode before template removal, the MIP sensor in the blank solution, and the MIP electrode after target rebinding (Figure 1a). The term “blank solution” refers to deionized (DI) water in which the MIP sensor is immersed after template removal. The signal from the MIP electrode before template removal increased as the O-PD concentration decreased from 2 mM to 0.5 mM. Electropolymerization forms a poly O-PD (pO-PD) layer, which is generally nonconductive [39]. Therefore, a higher concentration of O-PDs generates a thicker pO-PD layer, which inhibits electronic conductivity. Once the template is removed, cavities are formed within the pO-PD layer, creating an environment in which redox reactions can occur. This is reflected in the increased CV peak observed for the MIP electrode in the blank solution. The CV current increased when the MIP electrode was exposed to cortisol, which was unexpected, as the CV current was expected to decrease due to cortisol blocking the cavities. This can be attributed to several factors. First, the electropolymerized layer may be too thin to form cavities suitable for cortisol binding. Additionally, the increased signal may have been caused by the detachment of some pO-PD layers during washing. These factors may have contributed to the unexpected increase in the CV current. To address this issue and create thicker and more stable cavities, the number of CV cycles was further optimized.

The pO-PD layer is formed through the oxidation of the O-PD solution in an electrochemical cell, where CV is applied to facilitate the redox reaction [40]. The number of CV cycles directly influences the extent of the redox reaction and the resulting thickness of the pO-PD layer. An increased number of CV cycles were performed to create a thicker and more stable pO-PD layer (Figure 1b). When MIP electrode was fabricated using 15 CV cycles, the peak current showed similar tendency as MIP electrode fabricated using 9 CV cycles. However, after extracting the template, the peak current increased to 78.509 μA for the MIP electrode fabricated using 30 CV cycles. Subsequently, the peak current of MIP electrode after target rebinding slightly decreased to 76.976 μA as expected. Thus, the optimal number of CV cycles was determined to be 30. However, further optimization of the pH value and O-PD/Cortisol concentration was conducted due to insufficient peak current difference (1.533 μA) between the blank solution and 1 μM cortisol solution.

The effect of the pH of the electropolymerization solution is plotted in Figure 1c. The CV current peak of the MIP before removal decreased in the acidic environment, implying a thicker pO-PD layer. An acidic pH can facilitate the oxidation of O-PD monomers and promote the formation of the pO-PD layer during electropolymerization [41]. Therefore, a lower pH results in a higher polymerization rate, leading to a thicker and more compact pO-PD layer. However, it was observed that when the pH was lower than 5.2, the current peak between the blank solution and 1 μM cortisol solution was too small to distinguish concentration differences. On the other hand, the peak current difference between the blank solution and 1 μM cortisol solution increased from 1.533 to 4.85 μA as pH decreased from 7.4 to 5.2. Considering that a typical pH value used for O-PD electropolymerization is pH 5.2, we determined that pH 5.2 was the optimal condition for our experiment [26,42,43].

For further optimization, different O-PD and cortisol concentrations were tested as shown in Figure 1d. The peak current difference for different O-PD and cortisol concentrations of 2:2, 3.5:0.5, and 5:0.5 mM was 4.85, 9.713, and 4.231 μA, respectively. Among the tested combinations, the largest peak current difference was achieved when the O-PD concentration was 3.5 mM and the cortisol concentration was 0.5 mM. This result highlights the importance of the careful optimization of both O-PD and cortisol concentrations. If the template–monomer ratio is too low, there may be an insufficient number of template molecules to create a significant number of specific binding sites. However, if the ratio is too high, it may interfere with the cross-linking process during polymerization, resulting in an unstable MIP. By identifying the optimal parameters during the electropolymerization stage, it was possible to enhance the binding performance and selectivity of MIP for cortisol.

### 3.3. Template Removal Parameters Optimization

**Figure 2:**
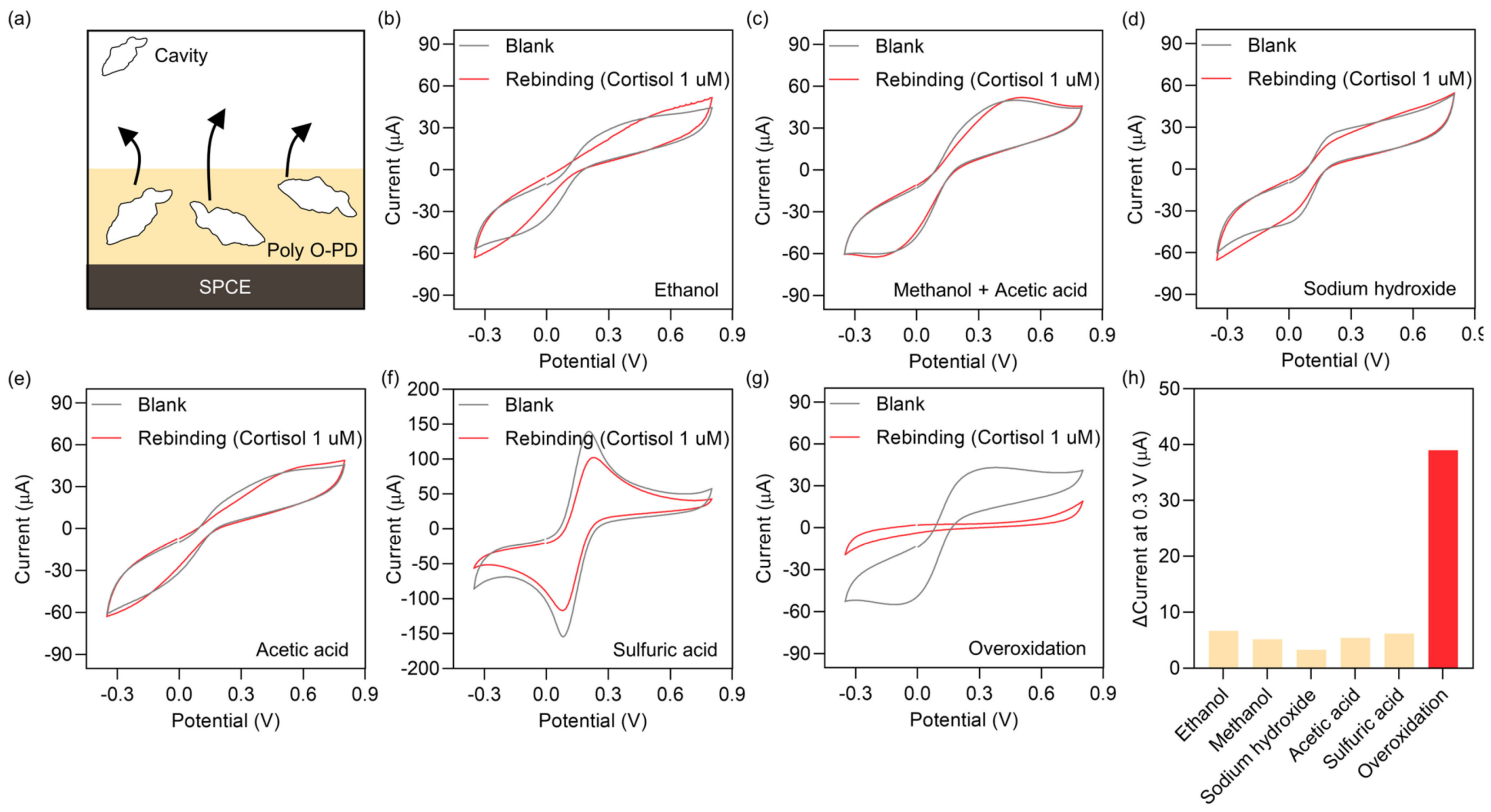
Optimization of template removal solution and method. (**a**) Schematic illustration of template removal process. (**b**–**g**) Cyclic voltammetry (CV) curves of the MIP sensor after template removal for each template removal method: (**b**) ethanol, (**c**) methanol with acetic acid (9:1 *v*/*v*), (**d**) sodium hydroxide 0.1 M, (**e**) acetic acid (10%), (**f**) sulfuric acid (10%), and (**g**) overoxidation. CV was conducted in a 5 mM [Fe(CN_6_)]^3−/4−^ solution containing 0.1 M KCL at pH 7, with a volume of 70 μL, scan range of −0.35~0.8 V, an E-step of 5 mV, and a scan rate of 50 mV/s. (**h**) The peak current differences (Δ*I_p_*) at 0.3 V between the MIP electrode in the blank solution and that after target rebinding.

After electropolymerization of the MIP layer, the template molecule (cortisol) was extracted from the polymer matrix, leaving behind cavities that were complementary in shape and size to the template molecule (Figure 2a) [32]. The template was removed by immersing the MIP sensor in a suitable solvent. Various solvents, including ethanol, methanol, acetic acid, sulfuric acid, and sodium hydroxide, have been used in previous studies. Another approach for template removal is overoxidation. Overoxidation involves the application of a high potential to the MIP sensor to induce the oxidation of the template molecule. Different solvent extraction methods and overoxidation techniques were tested to optimize the performance of the MIP sensor.

To assess the effect of the template removal method on the performance of the MIP sensor, CV curves were obtained for different scenarios. Figure 2b–f presents the CV curves for the MIP sensor in blank solution and after target rebinding, using various extract solutions (ethanol, methanol with acetic acid (9:1 *v*/*v*), sodium hydroxide 0.1 M, acetic acid (10%), and sulfuric acid (10%)) as well as the overoxidation technique (CV in PBS solution under following parameters: scan range of −0.35 to 0.7 V; E-step of 5 mV; and scan rate of 50 mV/s).

Figure 2b represents the CV curves of the samples extracted using ethanol. The current value around 0.3 V shows the oxidation of [Fe(CN_6_)]^4−^ to [Fe(CN_6_)]^3−^. The peak current around 0.3 V for the MIP sensor in the blank solution decreased when cortisol rebounded. However, the presence of the remaining redox signal suggests that not all cavities were filled after the rebinding process. This insufficient rebinding can be attributed to unsatisfactory extraction using ethanol. Similar trends were observed for methanol containing acetic acid (Figure 2c), NaOH (Figure 2d), and acetic acid (Figure 2e).

In contrast, when 10% sulfuric acid was used as the extract solution, a notable increase in the redox peak of [Fe(CN_6_)]^3−/4−^ was observed compared to other solvents. Furthermore, the difference between the oxidation peak potential (E_pa_ = 0.2 V) and reduction peak potential (E_pc_ = 0.08 V), Δ*E_p_*, was measured to be 120 mV. This value is smaller than the peak potential difference observed for the other extract solutions (i.e., $\Delta E_{p}$ of ethanol = 340 mV).

The Δ*E_p_* indicates the efficiency of electron transfer in a redox couple [44]. A larger $\Delta E_{p}$ indicates a hindered redox process, which in this case was hindered by the pO-PD layer. Therefore, an increased current peak and decreased $\Delta E_{p}$ cause pO-PD layer degradation, as 10% sulfuric acid is a strong acid that has the potential to damage the pO-PD layer.

In contrast, template removal via overoxidation resulted in excellent removal without damaging the pO-PD layer. Figure 2g shows the CV curves obtained when the overoxidation technique was used for the template removal. $\Delta E_{p}$ for the MIP sensor in blank solution was measured to be 330 mV, indicating that the MIP layer is stable after extraction. Moreover, after rebinding 1 μM of cortisol, the redox peak of [Fe(CN_6_)]^3−/4−^ was no longer detectable in the scan range of −0.35 to 0.8 V, suggesting that the cavities within the MIP layer were successfully filled by cortisol molecules, thereby hindering electron transfer.

To compare the efficiency of the template removal methods, the peak current differences (Δ*I_p_*) at 0.3 V between the MIP electrode in the blank solution and that after target rebinding is plotted in Figure 2h. This parameter serves as an indicator of the template removal efficiency and the ability of the MIP sensor to specifically bind cortisol. A higher difference in current indicated a more effective template removal process and a greater reduction in signal intensity after target binding, suggesting successful cavity filling by the target molecule. The differences in the current at 0.3 V were 6.668, 5.196, 6.167, 5.423, 3.298, and 39.038 for ethanol, methanol with acetic acid, sodium hydroxide, acetic acid, sulfuric acid, and overoxidation techniques, respectively. Among these methods, the overoxidation technique demonstrated superior template removal efficiency.

### 3.4. Rebinding Time Optimization

**Figure 3:**
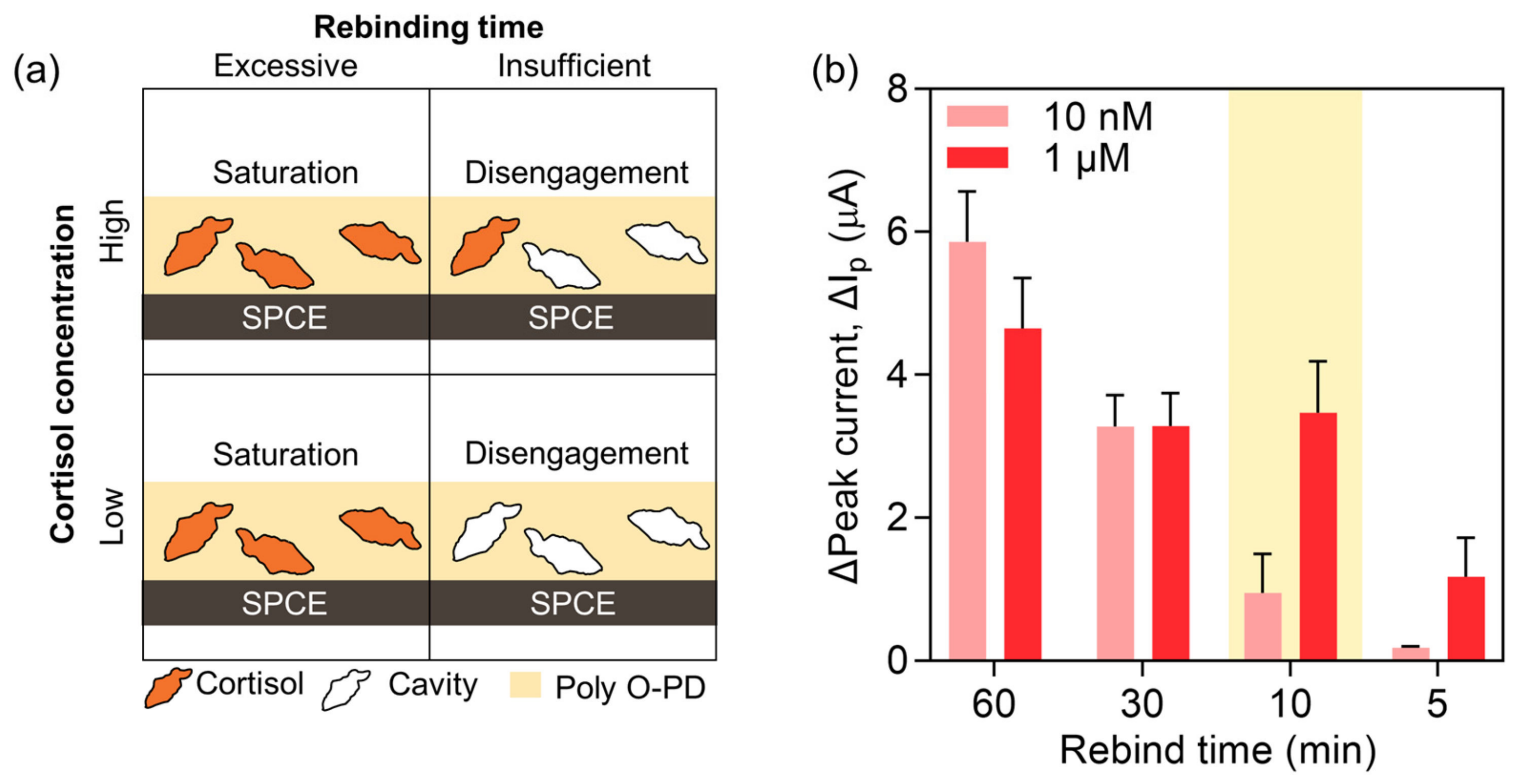
(**a**) Schematic illustration of relation between rebinding time and cortisol concentration. (**b**) Differential pulse voltammetry (DPV) peak current at different rebinding times. DPV parameters: a potential range of 0–0.8 V; a pulse amplitude of 50 mV/s; a pulse time of 10 ms; an E-step of 4 mV; and a scan rate of 50 mV/s.

Rebinding time plays a significant role in cortisol detection using MIP sensors [33]. An insufficient rebinding time can lead to a lack of interaction between the target molecules and the MIP sensor (Figure 3a). However, excessively long rebinding times may cause the target molecules to fully bind and reach saturation. In both cases, this hindered the ability of the sensor to distinguish the concentration differences. To achieve an appropriate detection range and maximize the sensor performance, it is essential to optimize the binding time, striking a balance between allowing sufficient interaction between the target molecules and the sensor and avoiding saturation effects.

We investigated the differential pulse voltammetry (DPV) responses between cortisol and the MIP sensor at different rebinding times (10 and 60 min). At a rebinding time of 60 min, we observed Δ*I_p_* of 6.364 and 5.147 μA at concentrations of 10 nM and 1 μM, respectively, compared with the blank solution. Unexpectedly, the Δ*I_p_* value at 10 nM was larger than that at 1 μM, suggesting a possible malfunction of the MIP sensor due to swelling [45,46]. O-PD-based MIPs require short reaction times to mitigate the potential swelling of aqueous solutions, and other studies mentioned in Table 1 have also utilized reaction times of less than 30 m for rebinding [28,29,30,34,35,36]. At a rebinding time of 30 min, we observed Δ*I_p_* of 3.277 and 3.280 μA for concentrations of 10 nM and 1 μM, respectively, compared with the blank solution. The Δ*I_p_* value at 10 nM and 1 μM showed only little difference, indicating that the malfunction of the MIP still exists due to long rebinding time. In contrast, at a rebinding time of 10 min, the Δ*I_p_* was 0.437 μA and 3.981 μA for concentrations of 10 nM and 1 μM, respectively, compared to the blank solution. At a rebinding time of 5 min, we observed Δ*I_p_* of 0.180 and 1.175 μA concentrations of 10 nM and 1 μM, respectively, compared with the blank solution. A rebinding time of 5 and 10 min successfully distinguished the concentration difference between 10 nM and 1 μM. However, the signal difference between 10 nM and 1 μM was larger at a rebinding time of 10 min. An insufficient rebinding time of 5 min may lead to a lack of interaction between the cortisol and the MIP sensor, which lead to little signal difference. Therefore, we confirmed that a rebinding time of 10 min was the most suitable for cortisol detection using the proposed MIP sensor. Finally, in the optimized conditions, each preparation step of the MIP sensor was subjected to electrochemical characterization analysis through CV (Figure S1).

### 3.5. Detection of Cortisol Using the MIP Sensor

**Figure 4:**
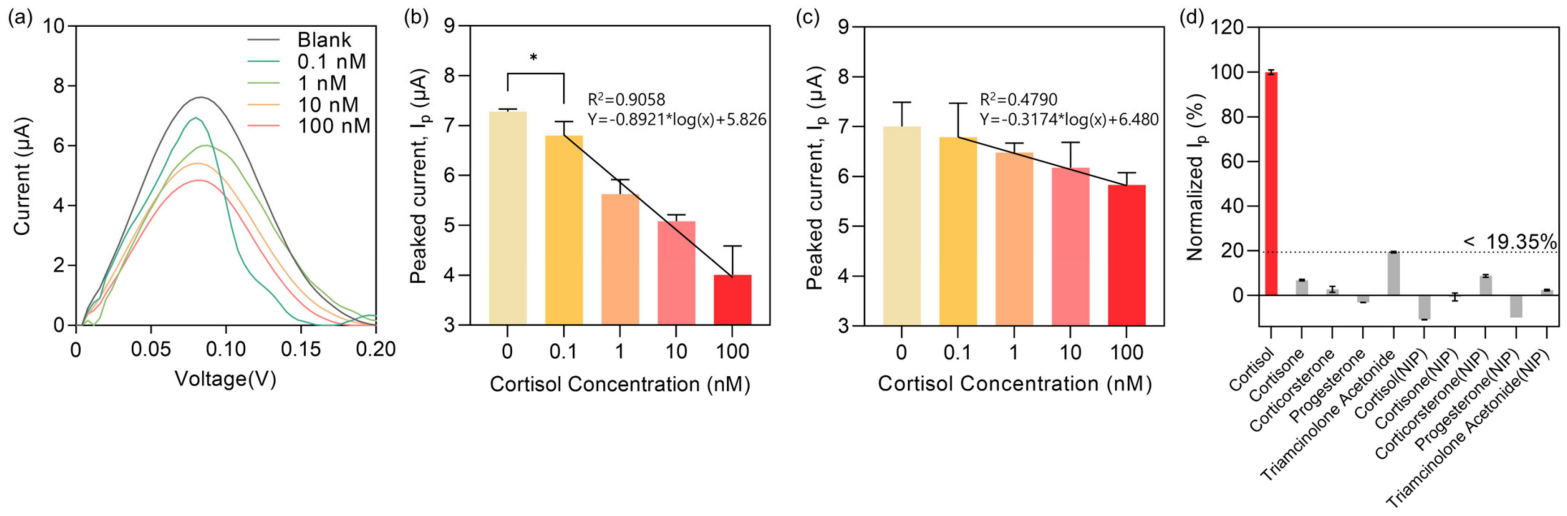
Sensitivity and selectivity analysis of the MIP sensor. (**a**) DPV curves and (**b**) corresponding peak current recorded at MIP sensor after rebinding different concentrations of cortisol (0.1–100 nM). (**c**) Peak current observed at NIP sensor after rebinding different concentrations of cortisol (0.1–100 nM). (**d**) Selectivity test of MIP and NIP sensor. DPV parameters: a potential range of 0–0.8 V; a pulse amplitude of 50 mV/s; a pulse time of 10 ms; an E-step of 4 mV; and a scan rate of 50 mV/s. As a *t*-test result, * p less than 0.05 was considered to be significant.

Under the optimized conditions, the DPV response of the MIP sensor to cortisol was recorded in 80 μL containing 10 mM PBS and 5 mM [Fe(CN_6_)]^3−/4−^. Figure 4a displays the DPV signal for the MIP sensor after rebinding different concentrations of cortisol (0.1–100 nM). The peak current around 0.8 V decreased proportionally with an increase in cortisol concentration. To quantitatively analyze the DPV responses, we plotted the peak current against the cortisol concentration (Figure 4b). The DPV detection was repeated three times, and the following linear equation was fitted:

(1) $$I_{p}=-0.8921log\left(C_{cortisol}\right)+5.826$$

where *I_p_* is the peak current around 0.8 V, and *C_cortisol_* is the concentration of cortisol. *I_p_*, depending on the cortisol concentrations, were 7.13 ± 0.19 (blank), 6.28 ± 0.62(0.1 nM), 5.53 ± 0.55 (1 nM), 4.68 ± 0.82 (10 nM), and 4.01 ± 0.58 (100 nM). As cortisol level in human saliva falls between 0.1 nM to 100 nM, the MIP sensor demonstrated outstanding sensitivity with a limit of detection (LOD) of 0.036 nM. The LOD was calculated using the equation LOD = 3.3 *σ*/*m*, where *σ* is the standard deviation of the DPV response of blank signal and *m* is the slope of the calibration curve.

A NIP was prepared without using a template, resulting in the absence of imprinted cavities specific for cortisol. Figure 4c illustrates the DPV peak current observed for the NIP sensor as a function of the cortisol concentration. The data were fitted to the following linear equation:

(2) $$I_{p}=-0.3174log\left(C_{cortisol}\right)+6.480$$

*I_p_*, depending on the cortisol concentrations, were 7.00 ± 0.49 (blank), 6.79 ± 0.68 (0.1 nM), 6.48 ± 0.19 (1 nM), 6.18 ± 0.51 (10 nM), and 5.83 ± 0.25 (100 nM). Comparatively smaller current differences were observed between concentrations in the NIP sensor, indicating nonspecific binding. Moreover, the slope of the linear equation of the NIP sensor was less than half that of the MIP sensor, indicating an effective imprinting effect induced by the MIP. From this result, we concluded that the optimized MIP sensor could effectively detect cortisol.

To confirm the selectivity of the MIP sensor, we conducted a comparative study of various steroid hormones with structures similar to that of cortisol, such as cortisone, corticosterone, progesterone, and triamcinolone (Figure 4d) [14]. The normalized peak current difference was calculated using the following equation: ((peak current of blank) − (peak current of other steroid hormones))/((peak current of blank) − (peak current of other cortisol)) × 100%. The concentration of each analyte was fixed at 100 nM. Significant differences were observed between the results of the tested substances and cortisol levels. These steroid hormones were also tested using the NIP sensor, and no significant peaks were found. The normalized current of the MIP sensor to cortisol was found to be five–ten times higher than that of other steroid hormones and the NIP sensor response. From these results, we confirmed that the MIP sensor could selectively detect cortisol compared with various steroid hormones.

To evaluate the reproducibility of the sensor, five different MIP sensors were fabricated, and each electrode was used to measure a concentration of 100 pM cortisol, as depicted in Figure S2a. The relative standard deviation (RSD) was calculated to assess the reproducibility of the measurements, and it was found to be 3.84%, indicating that the proposed sensor consistently provides reliable results across multiple sensor replicas.

Furthermore, the stability of the sensor was assessed by storing fabricated electrodes at room temperature in a petri dish for a duration of 3 days. The sensor’s response was monitored throughout the storage period (Figure S2b). The response remained within an acceptable range of 10.06% throughout the entire storage period. This demonstrates the sensor’s stability and its capacity to maintain consistent performance over a period of time.

## 4. Conclusions

In this study, we conducted a comprehensive investigation of the various parameters involved in the fabrication process of a bio-inspired MIP sensor for cortisol detection. Each parameter was carefully analyzed to establish a more consistent and effective method for MIP sensor fabrication. We successfully developed a robust cortisol detection strategy using a bio-inspired MIP sensor by optimizing different parameters, including monomer concentration, electropolymerization cycles, pH, monomer–template ratio, template removal technique, and rebinding time. Such optimization was crucial for achieving the desired thickness of the MIP layer, creating specific binding sites for cortisol, and enabling successful rebinding. Under the optimized conditions, the MIP sensor exhibited a proportional decrease in DPV peak currents with increasing cortisol concentration in the range of 0.1 to 100 nM. The sensor demonstrated excellent sensitivity with a limit of detection (LOD) of 0.036 nM. A comparison with a nonimprinted polymer (NIP) sensor confirmed the selectivity of the MIP sensor for cortisol. The cavity created within the MIP sensor exhibited specific binding affinity for cortisol, distinguishing it from other steroid hormones. Overall, this study provided crucial insights into the detection of cortisol using O-PD-based MIPs. The optimization of various fabrication parameters allowed for the development of a reliable and sensitive cortisol detection strategy. The findings of this study lay a foundation for future research in the field of cortisol detection and pave the way for advancements in MIP research.

## Data Availability

The authors confirm that the data supporting the findings of this study are available within the article.

## Supplementary Materials

The following supporting information can be downloaded at: https://www.mdpi.com/article/10.3390/biomimetics8030282/s1, Figure S1: Cyclic voltammetry (CV) curves of the proposed sensor at each modification step; Figure S2: Reproducibility and stability of the MIP sensor. (a) The normalized current peak for five different MIP sensor. (b) The normalized current peak for MIP sensor stored for 1 and 3 days. 100 pM of cortisol was measured for both reproducibility and stability test.
