# Supplementary material for: Bio-Inspired Molecularly Imprinted Polymer Electrochemical Sensor for Cortisol Detection Based on O-Phenylenediamine Optimization
Source: Biomimetics (Basel). 2023 Jul 1;8(3):282. doi: 10.3390/biomimetics8030282 (PMC10377510; doi:10.3390/biomimetics8030282)
Supplement: Supplementary file 1 [file biomimetics-08-00282-s001.zip › biomimetics-2451108-supplementary.pdf]

Supplementary information

Bio-Inspired Molecularly Imprinted Polymer Electrochemical Sensor for Cortisol Detection Based on O-Phenylenediamine Optimization

Minwoo Kim †, Daeil Park †, Joohyung Park * and Jinsung Park *

Department of Biomechatronic Engineering, College of Biotechnology and Bioengineering, Sungkyunkwan University, Suwon 16419, Republic of Korea; yegrina93@gmail.com (M.K.); pdi9907@gmail.com (D.P.)
* Correspondence: parkjoodori@gmail.com (J.P.); nanojspark@skku.edu (J.P.)
† These authors contributed equally to this work.

We analyzed the interfacial properties of each sensor preparation process using cyclic voltammetry (CV) (Figure S1). This interfacial characterization was performed under optimized conditions using a 5 mM $Fe(CN)_6^{3-/4-}$ electrolyte with 1 M KCl. Initially, in bare screen-printed carbon electrode (SPCE), an oxidation peak around 0.3 V and a reduction peak around 0 V were observed (yellow line). Subsequently, after performing electropolymerization for 30 cycles using the template solution containing cortisol and O-PD, a poly O-PD (pO-PD) layer was formed on the electrode surface along with cortisol. This led to a significant increase in surface resistance, making it difficult to observe the redox peaks of the electrolyte (black line). After removing cortisol from the template, the surface resistivity was restored, and the redox signal of the electrolyte became observable (red line). When introducing cortisol at a concentration of 1 μM, it specifically bound to the MIP sensor, thereby influencing the redox behavior of the electrolyte. This resulted in a further reduction in the observed redox signal (green line). These experimental results serve as evidence of the successful fabrication of the MIP sensor for cortisol sensing.

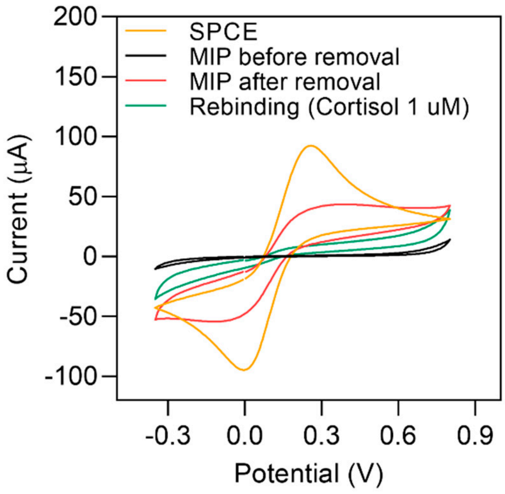

Figure S1. Cyclic voltammetry (CV) curves of the proposed sensor at each modification step.

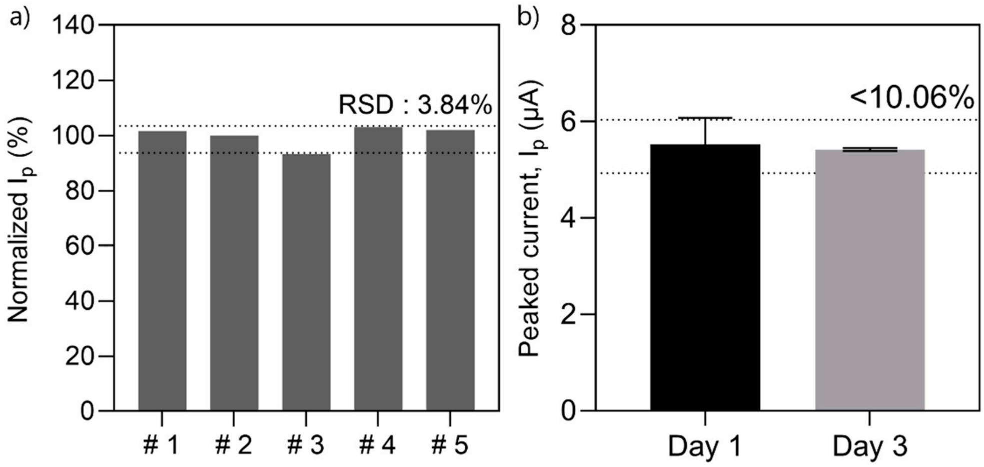

Figure S2. Reproducibility and stability of the MIP sensor. (a) The normalized current peak for five different MIP sensor. (b) The normalized current peak for MIP sensor stored for 1 and 3 days. 100 pM of cortisol was measured for both reproducibility and stability test.
